# Looking beyond meningococcal B with the 4CMenB vaccine: the *Neisseria* effect

**DOI:** 10.1038/s41541-021-00388-3

**Published:** 2021-10-29

**Authors:** Yara Ruiz García, Woo-Yun Sohn, Kate L. Seib, Muhamed-Kheir Taha, Julio A. Vázquez, Ana Paula S. de Lemos, Kumaran Vadivelu, Mariagrazia Pizza, Rino Rappuoli, Rafik Bekkat-Berkani

**Affiliations:** 1 GSK, Rockville, MD USA; 2 Institute for Glycomics, Griffith University, Gold Coast, QLD Australia; 3 Institut Pasteur, Paris, France; 4 National Centre of Microbiology, Instituto de Salud Carlos III, Madrid, Spain; 5 Adolfo Lutz Institute, São Paulo, Brazil; 6 GSK, Siena, Italy

**Keywords:** Protein vaccines, Bacterial infection

## Abstract

Infections with *Neisseria meningitidis* and *Neisseria gonorrhoeae* have different clinical manifestations, but the bacteria share up to 80–90% genome sequence identity. The recombinant meningococcal serogroup B (MenB) vaccine 4CMenB consists of four antigenic components that can be present in non-B meningococcal and gonococcal strains. This comprehensive review summarizes scientific evidence on the genotypic and phenotypic similarities between vaccine antigens and their homologs expressed by non-B meningococcal and gonococcal strains. It also includes immune responses of 4CMenB-vaccinated individuals and effectiveness and impact of 4CMenB against these strains. Varying degrees of strain coverage were estimated depending on the non-B meningococcal serogroup and antigenic repertoire. 4CMenB elicits immune responses against non-B meningococcal serogroups and *N. gonorrhoeae*. Real-world evidence showed risk reductions of 69% for meningococcal serogroup W clonal complex 11 disease and 40% for gonorrhea after 4CMenB immunization. In conclusion, functional antibody activity and real-world evidence indicate that 4CMenB has the potential to provide some protection beyond MenB disease.

## Introduction

**Fig. 1:**
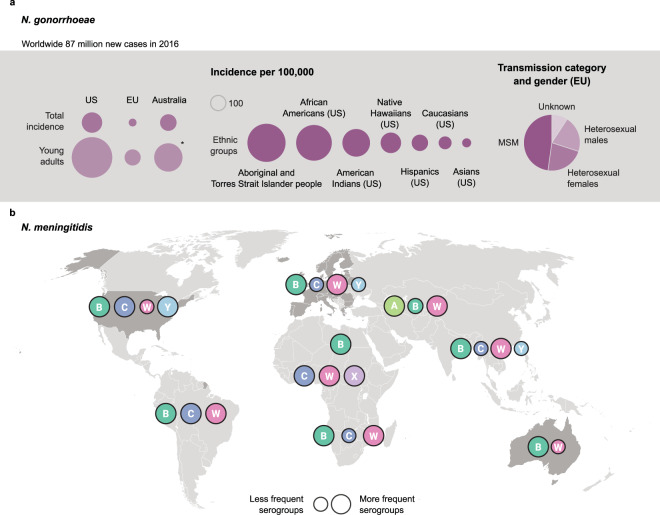
Incidence of gonorrhea^#8^ and global distribution of *Neisseria meningitidis* serogroups causing invasive meningococcal disease in 2019 (adapted from $). **a** Total incidence of gonorrhea in the United States (US), the European Union (EU), and Australia as well as incidence for young adults aged 20–24 years in 2018, incidence per ethnic group for Australia (Aboriginal and Torres Strait Islander people) and the US, and distributions per transmission category and gender for the EU. **b** The distribution of *N. meningitidis* serogroups and the countries where the incidence of gonorrhea is reported accurately in young adults (dark gray). A/B/C/W/X/Y meningococcal serogroup A/B/C/W/X/Y; MSM men who have sex with men. *Aged 20–29 years and data for 2017. ^#^https://kirby.unsw.edu.au/sites/default/files/kirby/report/KI_Annual-Surveillance-Report-2018.pdf; https://www.cdc.gov/std/stats18/tables/21.htm; https://www.cdc.gov/std/stats18/gonorrhea.htm; https://www.ecdc.europa.eu/sites/default/files/documents/gonorrhoea-annual-epidemiological-report-2018.pdf. ^$^https://www.who.int/images/default-source/health-topics/meningitis/map-serogroup-distribution-2019.png?sfvrsn=af422ab2_2.

The human pathogens *Neisseria meningitidis* and *Neisseria gonorrhoeae* are closely related, sharing up to 80–90% genome sequence identity^1–3^. However, infections with these bacteria have very different clinical manifestations^4,5^. *N. meningitidis* is present in the nasopharyngeal mucosa of healthy carriers and may infrequently lead to invasive meningococcal disease (IMD), a potentially life-threatening disease and a major cause of bacterial meningitis and septicemia worldwide^5,6^ (https://www.ecdc.europa.eu/en/meningococcal-disease/factsheet). The World Health Organization (WHO) has developed a global strategy to defeat meningitis by 2030 (https://www.who.int/initiatives/defeating-meningitis-by-2030). *N. gonorrhoeae* causes the sexually transmitted infection gonorrhea, which has a lower mortality rate than IMD but is more widespread^4–7^. In 2016, the number of new gonorrhea infections among individuals 15–49 years of age was estimated at 86.9 million worldwide, with variations in incidence between countries (Fig. 1a)^8^. The estimated prevalence of gonorrhea in the African Region in 2016 was 1.9% in women and 1.6% in men, while it was 0.3% in the European Region for both sexes^8^. As there is growing concern about the increasing incidence of gonococcal infections and the emergence of gonococcal antimicrobial resistance, the WHO has developed a global strategy to reduce gonorrhea by 90% by 2030 (https://apps.who.int/iris/bitstream/handle/10665/246296/WHO-RHR-16.09-eng.pdf;jsessionid=ADE613A231CC53A2301646DDCA496BB3?sequence=1).

For *N. meningitidis*, 12 disease-causing serogroups have been described so far based on the polysaccharide capsule. Of these, six meningococcal serogroups (MenA, MenB, MenC, MenW, MenX, and MenY) are responsible for almost all IMD worldwide, with serogroup distribution varying largely by region (Fig. 1b)^9,10^. Molecular typing schemes (such as multilocus sequence typing [MLST] and whole-genome sequencing [WGS]) allow a further classification of *N. meningitidis* into clonal complexes (cc), which are independent of meningococcal serogroups^9^. Several polysaccharide conjugate vaccines against MenA, MenC, MenW, and MenY, which are able to induce bactericidal antibodies directed against the polysaccharide capsule, are available^9^. Development of a vaccine against MenB has been more challenging as its polysaccharide capsule was proven to be poorly immunogenic because of the structural similarity with polysialic acid, a surface-exposed polysaccharide present on the human neural cell adhesion molecule^11–13^.

**Fig. 2:**
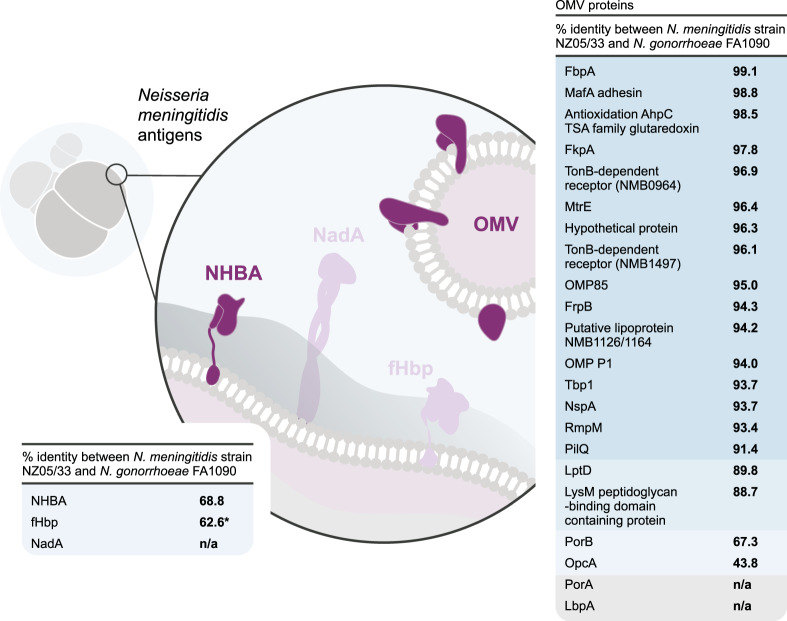
Main components of the 4CMenB vaccine and sequence similarity to gonococcal homologs (table adapted from Semchenko et al.^30^). NHBA neisserial heparin binding antigen, NadA *Neisseria* adhesin A, fHbp factor H binding protein, OMV outer membrane vesicle, FbpA ferric binding protein A, MafA multiple adhesin family A, AhpC alkyl hydroperoxide reductase C, TSA thiol-specific antioxidant, FkpA FKBP-type peptidyl-prolyl cis-trans isomerase, NMB *Neisseria meningitidis* strain MC58, MtrE outer membrane efflux protein, OMP outer membrane protein, FrpB Fe-regulated protein B, Tbp1 transferrin binding protein 1, NspA *Neisseria gonorrhoeae* surface protein A, RmpM reduction modifiable protein M, PilQ pili-associated protein Q, LptD lipopolysaccharide-assembly protein, LysM lysin motif, PorB porin, major OMP PIB, OpcA opacity protein A, PorA porin, serosubtype P1.4, LbpA lactoferrin binding protein A, n/a not available. *The gonococcal fHbp is not surface expressed.

The first vaccines developed against MenB were based on outer membrane vesicles (OMVs)^14,15^. However, these OMV vaccines (*VA-MENGOC-BC* [Finlay Institute] used in Cuba, *MenBvac* [Norwegian Institute of Public Health] in Norway, and *MeNZB* [Novartis] in New Zealand) were designed for specific strains, resulting in poor coverage across the diverse range of MenB strains^15^. Therefore, two protein-based vaccines against MenB (the four-component 4CMenB vaccine [*Bexsero*, GSK] and the bivalent fHbp2086 vaccine [*Trumenba*, Pfizer]) have been developed^16–19^. The fHbp2086 vaccine consists of equal amounts of two factor H binding protein (fHbp) variants belonging to subfamilies A and B, which were identified by biochemical approaches^17,19^. The 4CMenB vaccine contains three components that were identified by reverse vaccinology based on the complete genome sequence of a pathogenic reference MenB strain (MC58 strain)^18,20,21^: (1) the fHbp variant 1.1 (subfamily B) fused to the genome-derived *Neisseria* antigen (GNA) 2091, (2) the *Neisseria* adhesin A (NadA), and (3) the neisserial heparin binding antigen (NHBA) peptide 2 fused to GNA1030 (Fig. 2)^18,22^. Immunization with fHbp and NHBA fused to GNA2091 and GNA1030, respectively, resulted in increased bactericidal activity compared to immunization with the unfused proteins^18,22^. Besides these three recombinant surface-exposed protein antigens, the vaccine contains OMVs from the New Zealand strain NZ98/254 containing porin A (PorA) P1.4 (Fig. 2)^22^.

The 4CMenB vaccine offers broad protection against invasive MenB strains^22^ and is approved for use in >40 countries (GSK data on file). Each of the 4CMenB antigens induces bactericidal antibodies that mediate killing of strains based on antigen sequence similarity and level of expression^22^. It has been shown that the protein expression level^23^ and the number of expressed antigens^24^ may differ across meningococcal strains. Moreover, antibodies directed against fHbp, NadA, or NHBA can induce bactericidal killing in a cooperative manner^25–27^. In addition, antibodies elicited by 4CMenB immunization may recognize different epitopes on each of the fHbp, NadA, and NHBA antigens, further amplifying the bactericidal activity via this synergism^27^.

For *N. gonorrhoeae*, no effective vaccine has been developed yet, but there is increasing evidence that the 4CMenB vaccine may help protect against infection with gonococcal strains^28–30^. This is also the case for non-B meningococcal strains as most of them express the vaccine antigens^24,31^. The objective of this comprehensive review was therefore to outline and summarize the latest scientific evidence on genotypic and phenotypic similarities between 4CMenB vaccine antigens and their homologs, 4CMenB-induced immune responses, and protection against non-B meningococcal serogroups and *N. gonorrhoeae*.

## Mechanism of action of the 4CMenB vaccine

Meningococcal vaccines induce antibodies that could mediate bacterial killing by activating the classical complement pathway or by preventing factor H binding on the bacterial surface. However, these steps require a conserved amino acid sequence of the target antigen, which should also be present at a certain threshold density. Strains with a different sequence compared to some of the antigens present in the vaccine or with low expression of the target antigens may require multiple targets for binding of antibodies to activate the complement system. The multicomponent formulation of 4CMenB could address this need as multiple epitopes are present on each vaccine antigen and the antibodies induced by the vaccine components may act in a synergistic manner, thereby enhancing the bactericidal response of each antigen^22,25,27,32^.

The 4CMenB vaccine consists of three recombinant protein antigens, which are important for bacterial virulence. FHbp allows the bacteria to avoid complement killing and enables survival in blood. NHBA enables bacterial survival in blood and facilitates binding to epithelial cells, while NadA mediates binding to and invasion of human epithelial cells^22^. The OMV component provides protection to strains expressing the serosubtype P1.4 of PorA^22^.

Immunization with 4CMenB may provide protection against non-B meningococcal serogroups and gonococcal strains that contain one or more 4CMenB antigen genes or express vaccine antigens with close similarity and enough density^24,28–30^.

## Overview of findings associated with non-B meningococcal serogroups

Studies reporting strain coverage of 4CMenB against non-B meningococcal serogroups in more than ten countries were identified^31,33–42^ and are listed in Table 1. Results of the studies are described below based on the methodology used to predict coverage, including phenotypic assays (such as the meningococcal antigen typing system [MATS] or serum bactericidal antibody assays with human complement [hSBA]) and genomic approaches (such as genetic MATS [gMATS], *Bexsero* antigen sequence typing [BAST], or sequencing), as described in detail elsewhere^43^. The Meningococcal Deduced Vaccine Antigen Reactivity (MenDeVAR) Index has been recently developed to facilitate the interpretation of WGS data by practitioners. The MenDeVAR Index provides information on the presence and potential cross-reactivity of different meningococcal vaccine antigen variants^44^.

**Table 1:** Summary of the studies reporting (predicted) strain coverage of 4CMenB against non-B meningococcal serogroups.

Country	Strain coverage by 4CMenB (number of isolates tested)						Method used	Reference
	MenA	MenC	MenW	MenX	MenY	Total		
Canada		85.7% (7)	0% (10)	NA (1)	12.5% (16)		Sequencing	^33^
Spain		100%^a^ (4)	50%^a^ (2)	0%^a^ (2)	33%^a^ (6)	50% (14)	Sequencing	^34^
Australia		64% (50)	63% (27)	NA (1)	37% (30)	56% (108)	MATS	^35^
United Kingdom		5.7% (141)	0.2% (571)		2.9%^b^ (104)		BAST (≥1 exact match)	^36^
United Kingdom (2015–2016)		73.5%^c^ (34)	93.9% (197)		2.9% (104)		BAST (cross-reactive antigens)	^36^
Switzerland			15% (40)				gMATS	^37^
14 countries		17% (763)					Sequencing	^38^
England, Wales, Germany, France, Brazil		64% (80)	80% (35)		94% (32)	74% (147)	hSBA	^31^
England, Wales, Germany, France, Brazil		55.0% (80)	74.3% (35)		65.6% (32)	61.9% (147)	hSBA	^39^
Italy		18 and 45%^d^ (11)	100% (2)	100% (2)	100% (5)		hSBA	^40^
England, Wales			100% (6)				hSBA	^41^
Different countries	58.3–91.7% (4)						hSBA	Poster^e^
Africa, France				82%^a^ (11)			MATS and hSBA	^42^

*MenA/C/W/X/Y* meningococcal serogroup A/C/W/X/Y, *hSBA* serum bactericidal antibody assay with human complement, *MATS* meningococcal antigen typing system, *BAST* *Bexsero* antigen sequence typing, *gMATS* genetic meningococcal antigen typing system.

^a^Interpretation of published data.

^b^Data for 2015–2016.

^c^Of note, the publication reported 25/34 isolates (91.3%). Data for 2014–2015.

^d^18% for infant sera and 45% for adolescent sera; interpretation of the published data.

^e^Poster [P16] at the Meningitis and Septicaemia Conference; 2019; Biolchi, A., Giuliani, M., Tomei, S., Santini, L., Mori, E., Toneatto, D., La Gaetana, R., Giuliani, M.M. and Pizza, M.

### Coverage predictions of non-B meningococcal serogroups by the 4CMenB vaccine based on genotyping or phenotyping

In a Canadian study, 4CMenB vaccine coverage was assessed by sequencing 263 IMD isolates between 2009 and 2013 (MenB [*N* = 229], MenC [*N* = 7], MenW [*N* = 10], MenX [*N* = 1], and MenY [*N* = 16]), accounting for 75% of all IMD cases in Quebec. In this study, vaccine coverage was assumed for isolates with an exact match to either at least one of the vaccines antigens or fHbp variant 1 or NadA variants 1 or 2/3. For the non-B meningococcal serogroups, high strain coverage by 4CMenB antigens was predicted for MenC (85.7%), low coverage for MenY (12.5%), and no coverage for MenW (Table 1)^33^. In a Spanish study, allelic distribution of fHbp, NHBA, NadA, and PorA antigens was assessed in 82 invasive isolates (MenB [*N* = 68], MenC [*N* = 4], MenW [*N* = 2], MenX [*N* = 2], and MenY [*N* = 6]) collected between 2008 and 2013. It was estimated that 4CMenB offers protection against 50% of the non-B meningococcal strains (Table 1)^34^. MATS was used to assess the coverage of 4CMenB against non-B meningococcal serogroups (MenC [*N* = 50], MenW [*N* = 27], MenX [*N* = 1], and MenY [N = 30]) in an Australian study. Overall, the threshold for protection for at least one antigen was exceeded for more than half of the strains tested (MenC: 64% [95% confidence interval (CI): 46–86]; MenW: 63% [95% CI: 41–93]; MenY: 37% [95% CI: 27–43]) (Table 1)^35^. In a study in the United Kingdom, BAST was used to determine the distribution of 4CMenB antigens in the non-B meningococcal serogroups. Overall, 8/141 and 1/571 culture-confirmed isolates had an exact match to at least one 4CMenB antigen for MenC and MenW. For MenY, this was 3/104 culture-confirmed isolates in 2015–2016. The proportion of isolates with potentially cross-reactive variants of 4CMenB antigens was 25/34 for MenC in 2014–2015, 185/197 for MenW in 2015–2016, and 3/104 for MenW in 2015–2016 (Table 1)^36^. A Swiss study using gMATS reported that 6 out of 40 (15%) MenW isolates collected between 2010 and 2016 appeared to be covered by the 4CMenB antigen NHBA. All potentially covered isolates were assigned to MenW:cc22 (Table 1)^37^. A phylogenetic analysis of samples collected in 14 countries between 1970 and 2016 showed that 17% of 763 MenC isolates were potentially covered by the NHBA antigen of 4CMenB (Table 1)^38^.

Coverage predictions based on genotyping or phenotyping have some limitations. There was a wide variability in the predicted strain coverage by 4CMenB based on genotyping or phenotyping, depending on the strain tested (Table 1). The predicted strain coverage was 0% for MenX and ranged from 5.7 to 100%, from 0 to 93.9%, and from 2.9 to 37% for MenC, MenW, and MenY, respectively. It should also be noted that most studies included a limited number of isolates.

The main limitation of the genomic analyses is that only an exact match to the vaccine antigens is considered as a predictor of coverage, excluding the potential cross-immunity with antigen variants of <100% identity to the vaccine antigen. Additionally, the epidemiology of antigens expressed in *N. meningitidis* strains may vary across countries and regions as well as over time. As with other vaccines, this may also have an influence on the predicted coverage of 4CMenB^45^. On the other hand, MATS has limitations when used as predictor of coverage for non-B meningococcal strains as the MATS assay is established and calibrated based on MenB strains. Therefore, these analyses can be very informative but are limited in their ability to predict the real coverage.

### Immune response elicited by the 4CMenB vaccine against non-B meningococcal serogroups

#### Studies assessing bactericidal activity against multiple non-B meningococcal serogroups

The potential of 4CMenB to induce non-B meningococcal killing was assessed by hSBA on a panel of 147 isolates belonging to MenC (*N* = 76), MenW (*N* = 28), and MenY (*N* = 23) from England, Wales, Germany, and France and MenC (*N* = 4), MenW (*N* = 7), and MenY (*N* = 9) from Brazil^31,39^. Results showed that the overall strain coverage by pooled sera from 4CMenB-vaccinated infants—immunized with 4 doses at 2, 4, and 6 months of age with a booster dose at 12 months of age—was 74%, with 64% of MenC, 80% of MenW, and 94% of MenY isolates killed, while only 14% of isolates were killed by pooled pre-immune sera^31^. The overall strain coverage by pooled sera from adolescent vaccinees—immunized with 2 doses given 2 months apart—was 61.9%, with 55.0% of MenC, 74.3% of MenW, and 65.6% of MenY isolates killed, while only 7.5% of isolates were killed by pooled pre-immune sera (Table 1)^39^. The lower strain coverage by adolescent sera compared to infant sera is mainly caused by MenY strains. This may be due to differences in bactericidal assays as the infant sera were not heat inactivated and could therefore contain a higher amount of active complement and thus higher bactericidal titers^39^. The vaccine antigens responsible for protection were different between serogroups. Antigenic sequence data showed that protection against MenC was mainly mediated by antibodies elicited by NHBA, fHbp, and NadA while protection against MenW and MenY was mainly mediated by anti-NHBA antibodies. Anti-NadA antibodies could also play a role in the protection against Brazilian MenW isolates^31,39^. In a panel of 20 isolates from Italy (11 MenC, 2 MenW, 2 MenX, and 5 MenY), 18 and 45% of MenC isolates were killed by pooled sera from 4CMenB-vaccinated infants—immunized at 2, 4, and 6 months of age with a booster at 12 months of age—and adolescents—immunized with 2 doses with an interval of 2 months—respectively, and 100% of MenW, MenX, and MenY isolates were killed by both sera (Table 1)^40^. Immunization with 4CMenB is recommended for patients with complement deficiency as complement activation is a key step in the early immune response against IMD^32^ (poster [ESPID19-0684] at the European Society for Paediatric Infectious Diseases (ESPID)—37^th^ Annual meeting; 2019; Van Den Broek, B., de Jonge, M., de Groot, R., van de Ende, A., Langereis, J. and van der Flier, M.). These patients are also more prone to IMD caused by less common serogroups such as MenZ. A recent case report of a 6-year-old girl with complement deficiency showed that 4CMenB-induced immunoglobulin G was able to bind MenC and MenZ and increase complement activation, indicating a potential impact of 4CMenB on MenZ disease (poster [ESPID19-0684] at the European Society for Paediatric Infectious Diseases (ESPID)—37^th^ Annual meeting; 2019; Van Den Broek, B., de Jonge, M., de Groot, R., van de Ende, A., Langereis, J. and van der Flier, M.).

#### Studies assessing bactericidal activity against MenW

In a study evaluating the protection provided by 4CMenB in England and Wales, high hSBA titers (≥1:32) were observed in pooled sera from 4CMenB-vaccinated infants—immunized at 2, 3, and 4 months of age or 2, 4, and 6 months of age with a booster dose at 12, 18, or 24 months of age—against the hypervirulent MenW ST-11 cc11 (MenW:cc11) strain in all six tested isolates. Isolates possessed alleles for NadA and NHBA (albeit for different peptide variants), possibly mediating the cross-reactivity of 4CMenB against MenW:cc11 (Table 1)^41^. In another study in the United States (US) and Chile, hSBA titers ≥1:4 against a MenW:cc11 strain were reported in individual sera of 95% (95% CI: 75–100) of US adolescents (*N* = 20) immunized with 2 doses of 4CMenB^46^.

#### Studies assessing bactericidal activity against MenA

A recent study showed that 4CMenB may elicit functional immunity against MenA isolates, as MenA isolates (*N* = 4) were killed by sera from adolescents immunized with 2 doses of 4CMenB. Between 58.3 and 91.7% of MenA strains were killed by adolescent sera with an hSBA titer ≥1:4 (Table 1) and 45.8–83.3% with an hSBA titer ≥1:8 (poster [P16] at the Meningitis and Septicaemia Conference; 2019; Biolchi, A., Giuliani, M., Tomei, S., Santini, L., Mori, E., Toneatto, D., La Gaetana, R., Giuliani, M.M. and Pizza, M.).

#### Studies assessing bactericidal activity against MenX

The effect of 4CMenB on nine MenX isolates from several African countries (Niger [*N* = 7]; Chad [*N* = 1], and Burkina Faso [*N* = 1]) and two French MenX isolates was studied by MATS and hSBA. This study suggested 100% coverage by 4CMenB for the African MenX isolates, due to antibodies against fHbp or to a combination of anti-fHbp and anti-NHBA antibodies. The coverage for the two French isolates, which were genetically different from the African isolates, was 0% (Table 1)^42^.

#### Limitations of hSBA in predicting coverage

Although studies assessing bactericidal activity can give an indication of coverage, the hSBA assay has some limitations. Due to wide genetic diversity and temporal changes, data from a limited number of reference strains cannot be extrapolated to all the strains of the same serogroup. However, testing of a large number of strains is not feasible as this would require large amounts of sera from immunized individuals, as well as different sources of complement. The limited availability of suitable human complement poses an additional challenge as it should be collected from individuals who do not have antibodies against meningococcal strains^43^. The vaccine was licensed based on clinical trials that gathered safety and immunogenicity data using hSBA assays. Evidence of effectiveness was expected to be collected after licensure^47^.

### Real-world evidence (RWE) of 4CMenB vaccination against non-B meningococcal serogroups

To overcome the limitations of the previously described methods, real-world effectiveness and impact data are important. However, these data can only be collected after broad implementation of a vaccine^43,47^.

A study in England estimated the impact of 4CMenB immunization on MenW disease in age cohorts fully eligible to receive the vaccine in the context of the national infant immunization program (NIP). In 2015, the United Kingdom was experiencing a national outbreak of MenW:cc11, possessing a NadA antigen that is known to be cross-reactive with the 4CMenB NadA antigen and an NHBA antigen that could be a potential target for 4CMenB. The MenACWY vaccine was thus not administered to infants or toddlers in the United Kingdom. During the 4 years after the introduction of 4CMenB vaccination in the NIP in 2015, an estimated 98 MenW cases (95% CI: 34–201) were prevented in vaccine-eligible children. It should be noted that the estimated indirect impact of the adolescent MenACWY program through reduction of carriage in adolescents may have been responsible for preventing more MenW cases in infants than the protection provided by 4CMenB vaccination. However, the authors stated that both 4CMenB and MenACWY vaccination strategies are individually effective for the prevention of MenW:cc11 disease. Model estimates showed that the number of MenW cases was reduced by 69% (adjusted incidence rate ratio: 0.31 [95% CI: 0.20–0.67]) in 4CMenB-eligible children, regardless of their vaccination status. This study provided the first RWE on the protection elicited by 4CMenB against MenW disease, with 89% of strains being MenW:cc11^48^. These results should be interpreted with care as protection conferred by 4CMenB against all MenW strains will depend on the presence and degree of expression of vaccine antigens on the meningococcal surface. In a Portuguese RWE study in infants, children, and adolescents, the vaccine effectiveness was 78% for IMD caused by any serogroup. It should be considered that the majority of IMD in Portugal was caused by MenB during the study period (2014–2019)^49^.

## Overview of findings associated with *N. gonorrhoeae*

**Fig. 3:**
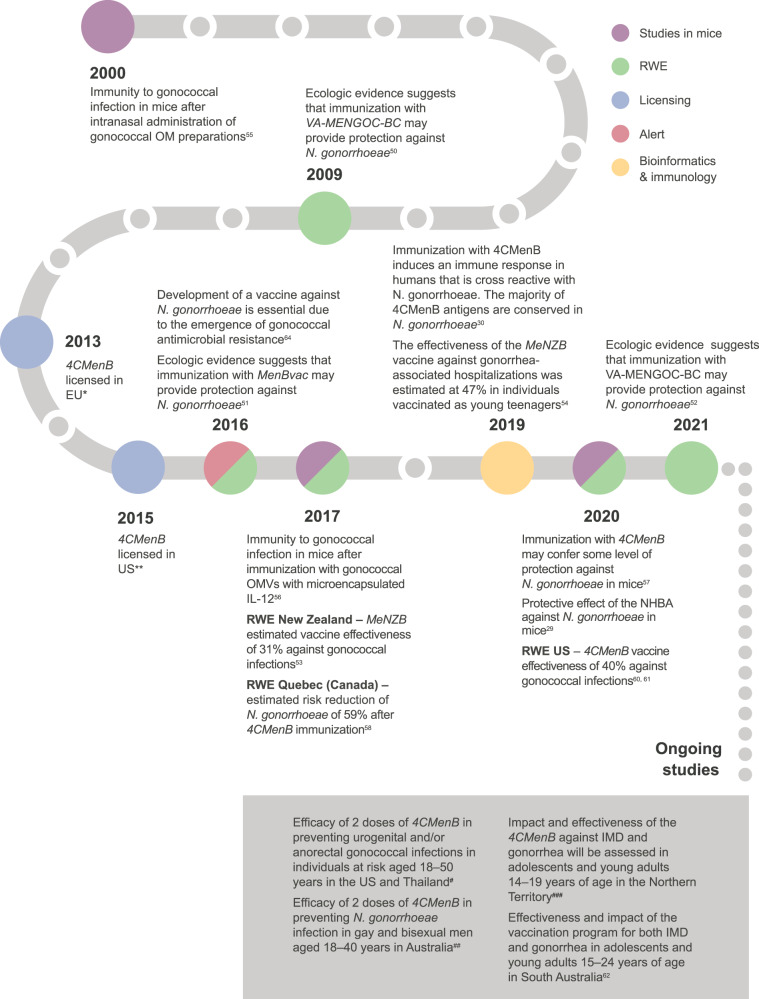
Timeline of findings and ongoing studies associated with *Neisseria gonorrhoeae*^29,30,50–58,60–62,64^. OM outer membrane, RWE real-world evidence, EU European Union, US the United States, OMV outer membrane vesicle, IL-12 interleukin 12, NHBA neisserial heparin binding antigen, IMD invasive meningococcal disease. *https://www.ema.europa.eu/en/documents/product-information/bexsero-epar-product-information_en.pdf. **https://www.fda.gov/media/90996/download. ^#^ClinicalTrials.gov: NCT04350138. ^##^ClinicalTrials.gov: NCT04415424. ^###^ClinicalTrials.gov: NCT04398849.

To date, a vaccine against gonorrhea is not yet available. A timeline of the findings and ongoing studies associated with *N. gonorrhoeae* is presented in Fig. 3.

### Protection provided by OMV vaccines against *N. gonorrhoeae*

Ecological evidence from Norway and Cuba was the first to indicate that MenB OMV vaccines may offer protection against *N. gonorrhoeae* as surveillance data suggested a reduction of gonorrhea cases^50–52^. These results were confirmed by a retrospective case–control study in New Zealand showing that the *MeNZB* vaccine had an estimated vaccine effectiveness against gonorrhea of 31% (95% CI: 21–39) in a population that received 3 doses of the vaccine and was up to 20 years of age at the time of vaccination. The effect of the vaccine may, however, differ depending on the *N. gonorrhoeae* strain as strains vary widely^53^. Another retrospective study conducted in New Zealand estimated that the effectiveness of the *MeNZB* vaccine against gonorrhea-associated hospitalizations was 47% (95% CI: 18–66) in individuals vaccinated as young teenagers^54^.

### Genomic similarities between the 4CMenB vaccine antigens and *N. gonorrhoeae* proteins

A recent study confirmed the absence of a gene for NadA and the presence of genes for fHbp, NHBA, GNA1030, and GNA2091 in *N. gonorrhoeae* isolates^30^. Since fHbp, GNA1030, and GNA2091 are not surface exposed in *N. gonorrhoeae*, NHBA is believed to be the only recombinant antigen of 4CMenB that may induce protection against *N. gonorrhoeae*. Semchenko et al.^30^ used both bioinformatic and immunological approaches to provide further evidence for 4CMenB-induced protection against *N. gonorrhoeae*. Besides a high level of amino acid sequence identity between most of the major 4CMenB OMV proteins and *N. gonorrhoeae* homologs (Fig. 2), they demonstrated that OMV-induced antibodies recognized gonococcal proteins. Results reported by Marjuki et al. are in line with these findings as they indicate that some outer membrane proteins (OMPs) present in the 4CMenB OMV have ≥93% sequence similarity with *N. gonorrhoeae* OMPs, such as OMP85 (BamA), NspA, MtrE, and MetQ, and may contribute to protection against *N. gonorrhoeae*^28^. Overall, the high sequence identity for NHBA proteins in *N. meningitidis* and *N. gonorrhoeae* suggests a protective effect induced by 4CMenB immunization on top of the one provided by the OMV vaccine alone^30^.

### Immunogenicity of gonococcal OMVs and the 4CMenB vaccine against gonorrhea in mice

Intranasal administration of lithium chloride-extracted gonococcal outer membrane preparations to female mice resulted in an accelerated clearance of *N. gonorrhoeae* compared to the control group. It also resulted in the detection of gonococcal-specific antibodies in the sera of the immunized mice^55^. Accelerated clearance of *N. gonorrhoeae* and induction of antibodies were also reported in a study investigating intravaginal administration of gonococcal OMVs with microencapsulated interleukin-12 in female mice^56^.

A recent study showed that administration of 4CMenB to mice with an *N. gonorrhoeae* genital tract infection resulted in accelerated clearance and reduction of the number of bacteria as compared to treatments with aluminum or phosphate-buffered saline. Additionally, immunization with 4CMenB resulted in the induction of antibodies able to recognize *N. gonorrhoeae* OMPs. These results imply that immunization with 4CMenB may confer some level of protection against *N. gonorrhoeae*^57^.

### RWE of 4CMenB vaccination against *N. gonorrhoeae*

A 4CMenB vaccination campaign conducted in the Saguenay-Lac-Saint-Jean region of Quebec (Canada) resulted in an estimated risk reduction for *N. gonorrhoeae* of 59% (95% CI: −22 to 84; *P* = 0.1)^58^. The campaign started in 2014 in response to an increase in IMD incidence starting in 2003 and targeted individuals between 2 months and 20 years of age. The immunization schedule varied with age with 4 doses for infants aged 2–5 months, 3 doses for infants aged 6–11 months, and 2 doses for individuals ≥12 months of age. The interval between doses was at least 2 months^59^. In the post-vaccination period, the number of *N. gonorrhoeae* cases among vaccinated individuals aged 14–20 years declined, while an increase was observed in those ≥21 years of age (unvaccinated individuals). The sexually transmitted infection *Chlamydia trachomatis* monitored during the same time period increased among individuals of both age groups. Of note, the absence of statistical significance was likely due to the low study power^58^.

In addition, a retrospective case–control study conducted in the US to assess the potential of 4CMenB to prevent gonococcal infections reported a vaccine effectiveness of 40% (95% CI: 25–53) in individuals aged 16–23 years who had received 2 doses of the vaccine 1–6 months apart^60,61^.

### Ongoing studies assessing immunogenicity, effectiveness, and impact of 4CMenB against *N. gonorrhoeae*

A phase II, randomized, observer-blind, placebo-controlled trial is being conducted in the US and Thailand to assess the efficacy of two doses of 4CMenB in preventing urogenital and/or anorectal gonococcal infections in individuals at risk. The target enrollment is 2200 participants and is ongoing. Both men and women, 18–50 years of age, can enroll and the estimated study completion date is August 1, 2023 (ClinicalTrials.gov: NCT04350138). In Australia, a phase III, randomized, double-blind, placebo-controlled trial assessing the efficacy of two doses of 4CMenB in preventing *N. gonorrhoeae* infection in gay and bisexual men (including men [cis and trans], trans women, and non-binary people who have sex with men) is planned to start in April 2021. The target enrollment is 730 men (cis and trans) aged 18–40 years and the estimated completion date is February 2024 (ClinicalTrials.gov: NCT04415424).

In the Northern Territory (Australia), the impact and effectiveness of the 4CMenB vaccine (as part of the Northern Territory immunization program) against IMD and gonorrhea will be assessed in adolescents and young adults 14–19 years of age. The target enrollment is 7100 participants and the estimated completion date is December 31, 2024 (ClinicalTrials.gov: NCT04398849). Another study is being conducted in South Australia in adolescents and young adults 15–24 years of age to determine the effectiveness and impact of the 4CMenB vaccination program on both IMD and gonorrhea over a period of 3 years^62^.

## Discussion

4CMenB components are present and conserved in several *N. meningitidis* and *N. gonorrhoeae* strains^3,24^. Recent results suggest that 4CMenB generates bactericidal immunity against non-B meningococcal serogroups and may provide some protection against MenW disease^31,33–42,48^. Varying degrees of strain coverage have been estimated due to variable conservation of 4CMenB components^31,33–42^. The first study generating RWE on the effect of 4CMenB against MenW disease has recently been published and estimated a 69% reduction of MenW:cc11 cases in 4CMenB-eligible children, regardless of vaccination status^48^. As immunity depends on several aspects, such as the presence, the surface expression, and the sequence diversity of the antigens as well as the antigen accessibility to bactericidal antibodies, the potential level of protection afforded may vary depending on the strain studied^41^. In this comprehensive review, varying degrees of strain coverage of non-B meningococcal serogroups are reported depending on the serogroup, geography, and techniques used.

Preliminary studies indicate that 4CMenB may also provide protection against *N. gonorrhoeae* infections^28,30,57^, and RWE studies estimated a risk reduction for *N. gonorrhoeae* of 59% (not statistically significant) following a vaccination campaign in Quebec^58^ and a vaccine effectiveness of 40% in individuals who had received 2 doses of 4CMenB 1–6 months apart in the US^60,61^. Further research regarding the recognition of gonococcal antigens by 4CMenB-induced antibodies is needed. Clinical trials specifically designed to assess immunity and effectiveness are ongoing in several countries (ClinicalTrials.gov: NCT04415424, NCT04350138, NCT04398849, and Marshall et al.^62^) and will help to inform on the full potential for 4CMenB vaccination to help reduce gonorrhea infections.

Future genomic and proteomic characterizations of IMD and gonorrhea isolates will provide information on the molecular basis of the underlying broad strain coverage, while informing the formulation of future non-capsular meningococcal vaccines and support decisions regarding prevention and immunization programs.

Some of the limitations of this review are linked to the methods used in the studies: (1) gMATS and MATS are specifically designed and validated for MenB, not for other serogroups nor for *N. gonorrhoeae*, and do not consider the potential synergies between antibodies targeting the recombinant antigens and the minor OMV antigens, (2) genotype- and phenotype-based approaches are not designed to predict synergistic immunogenic effects of antibodies binding simultaneously to different antigens. For these reasons, the true coverage rate is likely to be higher than the one estimated by genotyping and phenotyping. This underestimation has also been reported for MenB strains^43^. Currently available results of studies evaluating the potential protection conferred by 4CMenB against *N. gonorrhoeae* should be interpreted within context as they were based on observational and ecological studies, and vaccine efficacy has not yet been determined. The controls (*C. trachomatis* infections) used in these studies may also not be appropriate to assess potential protection as infected individuals may be more prone to infection with other sexually transmitted diseases due to tissue damage^63^. Moreover, the duration of protection still has to be determined. Serological studies are also difficult to interpret in the absence of a correlate of protection, as is the case for meningococci. The assessment of the potential protection conferred by 4CMenB is further challenged by the fact that additional studies are still needed to fully understand the natural immune responses against gonococcal infections. A further limitation could be the potential differences in mechanism of protection against *N. gonorrhoeae*, causing local infections, and *N. meningitidis*, causing systemic infections. A local infection can occur at different anatomical sites and makes the testing, reporting, notification, and surveillance of gonorrhea more challenging than for IMD as points of testing do not usually assess infection at all anatomical sites and some gonorrhea cases may be missed, thereby allowing further spread of the infection.

The strengths of this review are the well-characterized assays used in the included studies and the qualitative and quantitative description of the potential protection of 4CMenB against non-B meningococcal serogroups and *N. gonorrhoeae*. Moreover, this review allows a better understanding of the full potential of multicomponent meningococcal vaccines and their mechanism of action and may serve as a guide for future vaccine development.

A plain language summary of our findings and their clinical relevance is presented in Box 1.

In conclusion, this review assessed the existing evidence on the effect of 4CMenB, a multicomponent vaccine, on IMD caused by different meningococcal serogroups and on gonorrhea. Several studies using different methodologies to predict strain coverage of 4CMenB against non-B meningococcal serogroups and RWE showed that the 4CMenB vaccine could potentially offer some level of protection against non-B meningococcal serogroups and *N. gonorrhoeae*.

Box 1 Plain language summary **What is the context?**

- *Neisseria meningitidis* is a bacterium that causes invasive meningococcal disease (IMD), a potentially life-threatening infection. The bacteria are classified into different types (i.e., serogroups) based on components they have in common. Six serogroups (A, B, C, W, X, and Y) are responsible for most meningococcal infections.
- *Neisseria gonorrhoeae* is a bacterium that causes gonorrhea, a sexually transmitted infection. Its genes are 80–90% identical to *N. meningitidis*. An effective vaccine against gonorrhea has not yet been developed.
- The 4CMenB vaccine (*Bexsero*, GSK) was designed to help prevent IMD caused by serogroup B and consists of four components. Some of these components may also be present in the other serogroups that cause IMD (such as A, C, W, X, and Y, so called non-B serogroups) and in *N. gonorrhoeae*.
- We summarized the available information demonstrating the ability of the 4CMenB vaccine to help protect against non-B serogroups and *N. gonorrhoeae*, as well as ongoing studies to learn about the impact of the 4CMenB vaccine on *N. gonorrhoeae*.

**What does this review highlight?**

- Our review showed that
  ○ The 4CMenB vaccine demonstrated an effect against non-B serogroups and gonococcal infections if the bacteria’s components are similar to one or more components of the vaccine.
  ○ Antibodies produced by individuals vaccinated with 4CMenB can lead to the killing of non-B serogroups.
  ○ The risk of IMD caused by a subset of serogroup W was reduced by 69% after introduction of the 4CMenB vaccine in England. Indirect protection of infants provided by MenACWY vaccination of adolescents is expected to be higher, but both strategies are individually effective.
  ○ Vaccination with 4CMenB reduced the risk of gonorrhea by 40% in adolescents and young adults in the United States. Efficacy of the 4CMenB vaccine against gonococcal infections is under investigation.

**What is the take-home message?**

- There is increasing evidence that the 4CMenB vaccine might also reduce meningococcal disease caused by non-B serogroups and gonococcal infections.
- Understanding the potential of this multi-component vaccine can inform future decisions about vaccine development and vaccination programs for IMD and gonorrhea.

## Methods

### Literature search

A literature search was conducted in PubMed, EMBASE, Google, and conference proceedings for English publications using the following search terms (1) *Bexsero* or 4CMenB or MenB-4C and (2) multilocus sequence typing or MLST or whole genome sequencing or WGS or genotype similarity or phenotype similarity or sequence similarity or serum bactericidal antibody assays with human complement or hSBA or immunogenicity or immune response or cross-protection or cross-reactivity and (3) *N. gonorrhoeae* or *N. meningitidis*. No specific time period was identified.

The inclusion criteria were: (1) studies reporting genomic/proteomic similarities between non-B *N. meningitidis* and vaccine antigens and between *N. gonorrhoeae* and vaccine antigens, (2) strain coverage and immunogenicity studies of 4CMenB against non-B meningococcal isolates and *N. gonorrhoeae*, and (3) effectiveness and impact of 4CMenB against non-B IMD and gonorrhea infection. To ensure an objective review of the literature and to reduce bias, we first undertook a preliminary search to identify published articles relevant to the field of interest. Articles and abstracts to include were then selected based on titles and abstracts. Studies reporting protection of 4CMenB against MenB disease were excluded.

### Methods to assess the potential of 4CMenB to protect against non-B serogroups and gonococcal infections

**Fig. 4:**
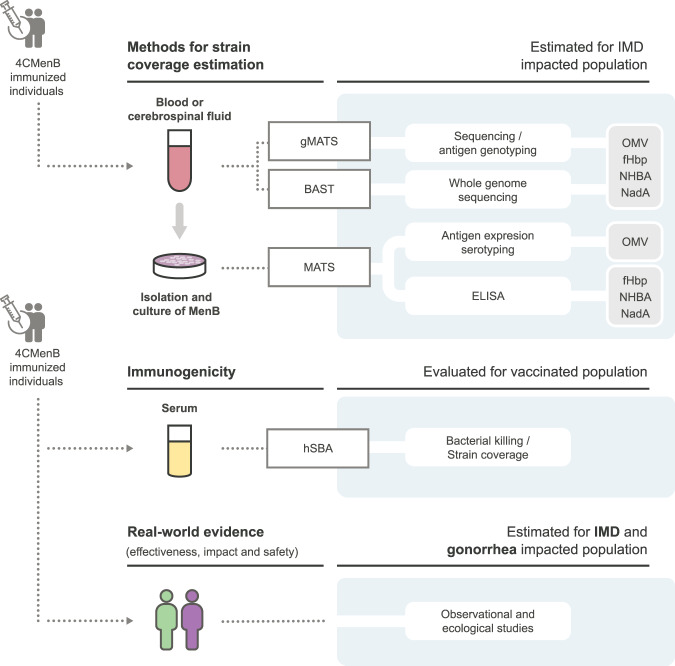
Schematic representations of methods currently used to predict strain coverage, assess immunogenicity, effectiveness, and impact of the 4CMenB vaccine. IMD invasive meningococcal disease, MenB meningococcal serogroup B, MATS meningococcal antigen typing system, gMATS genetic MATS, BAST *Bexsero* antigen sequence typing, ELISA enzyme-linked immunosorbent assay, OMV outer membrane vesicle, fHbp factor H binding protein, NHBA neisserial heparin binding antigen, NadA *Neisseria* adhesin A, hSBA serum bactericidal assay with human complement.

The methods used to assess immunogenicity and estimate strain coverage by 4CMenB are presented in Fig. 4 and explained in further detail elsewhere^43^.

## Data Availability

No new datasets were generated or analyzed for this publication.
